# The Present and Future of Robotic Surgery in Breast Cancer and Breast Reconstruction

**DOI:** 10.3390/jcm14062100

**Published:** 2025-03-19

**Authors:** Brett Allen, Alexis Knutson, Noama Iftekhar, Casey Giles, Jarrell Patterson, Joshua MacDavid, Richard Baynosa

**Affiliations:** Department of Plastic Surgery, University of Nevada, Las Vegas, NV 89102, USA; alexis.knutson@unlv.edu (A.K.); noama.iftekhar@unlv.edu (N.I.); casey.giles@unlv.edu (C.G.); jarrellpatterson3@gmail.com (J.P.); joshua.macdavid@unlv.edu (J.M.)

**Keywords:** robotic breast reconstruction, DIEP, latissimus dorsi, da Vinci, plastic surgery, robotic surgery

## Abstract

**Background:** Breast cancer is the second most common cancer in women with an improving mortality rate and a growing need for reconstruction following oncologic resection. Advancements in robotic surgery and minimally invasive techniques have offered refinement to traditional open techniques of flap harvest for reconstruction, particularly regarding improved donor site morbidity. **Methods:** The literature review was based on a PubMed database search using the keyword “Robotic breast reconstruction” in conjunction with the Boolean operators “Flap”, “Latissimus”, and “DIEP” to specify the search. In total, 106 results were generated, which were then manually reviewed and condensed for a comprehensive stance on the current status, technique, variations, and outcomes for robotic breast reconstruction. **Results:** Robotic technique has been described for the latissimus dorsi (LD) and deep inferior epigastric perforator (DIEP) flaps for breast reconstruction. For LD, robotic flap harvest reduces donor site morbidity, incisional length, and hospital length of stay, with similar complication rates for seroma/hematoma/infection, and longer operative times. Robotic LD procedures have been described in conjunction with single-site nipple-sparing mastectomy and flap elevation leading to a full minimally invasive resection and reconstruction from one lateral incision. Robotic DIEP harvest offers a considerably smaller fascial incision/rectus muscle dissection and has a comparable complication rate to traditional techniques with shorter hospital length of stay, and improved pain, at the expense of longer operating times. Data on hernia/bulge reduction from robotic techniques is limited and not yet available. **Conclusions:** Robotic breast reconstruction offers great potential for improving breast reconstruction in terms of donor site morbidity, length of incision, hospital length of stay at the cost of longer operating times, and increased technical skill/specialization, but it has yet to be proven on a large scale with long-term outcome data. Multi-center, prospective clinical data and trials are needed to help elucidate the potential for equivalence and superiority of the minimally invasive approach compared to standard open techniques, but the future is promising for robotic surgery in breast cancer and breast reconstruction.

## 1. Introduction

One of the most common reconstructive challenges faced in the modern age is that of breast reconstruction. While the lifetime risk of developing breast cancer is about one in eight for women in the US, the mortality rate has reduced by 58% through improved screening and treatment advancements over the last half century [1]. With the rising frequency of breast cancer operations/oncologic resections, the need for restorative surgeries is growing as well. In the US, the long-term trend of breast cancer has decreased mortality and decreased severity at presentation, largely attributable to improved screening, treatment algorithms, and detection [1,2]. Oncologic breast surgery has evolved from the early days of radical mastectomy, to modified radical mastectomy, to skin-sparing mastectomy, to nipple-sparing mastectomy [3]. Focus on preserving appearance and contour has been paramount in the evolving trends of breast surgery. In the US, access to breast reconstruction is assured by the Women’s Health and Cancer Rights Act (WHCRA) of 1998 [4]. Breast reconstruction techniques have evolved with two main categories arising: implant-based reconstruction and autologous flap-based reconstruction. Implant-based reconstruction is the more traditional of the two, with many similar concepts to cosmetic breast augmentation. A major pitfall with implant-based reconstruction is the drawbacks associated with a radiated field. There is a higher rate of scar tissue formation, capsular contracture, and long-term deformity and the optimal reconstructive strategy is delayed autologous reconstruction [5]. Autologous flap-based reconstruction overcomes many of these flaws by bringing the patient’s vascularized non-radiated tissue/skin into the operative/radiated field. Autologous reconstruction decreases the risk of capsule formation/contracture and implant rupture/replacement, can provide additional skin, and can be neurotized to restore a degree of sensation. Drawbacks to autologous reconstruction include selective patient candidacy based on habitus, donor site anatomy, morbidity/complications, longer operative times, and complications associated with pedicle dissection and microsurgery [6]. Autologous reconstruction is favored in breast cancer patients having undergone radiation but is selective and contingent on the individual characteristics of each patient. 

There are two classes of flap-based reconstruction—free flaps and pedicled flaps. Pedicled flaps are vascularized tissue elevated from a nearby body part and inset to the desired site with their original blood supply intact. Free flaps are tissue from another body part with the blood vessels being transected and re-anastomosed in a new connection [7]. Latissimus dorsi (LD) and deep inferior epigastric perforator (DIEP) flaps have risen as the most common flaps for breast reconstruction. The LD flap is typically harvested as a pedicled flap in conjunction with an implant but can also be used by itself for small-volume breast defects in the case of partial mastectomy/quadrantectomy or in the case of Poland syndrome which includes agenesis of the pectoralis muscle and chest wall deformity [8,9]. The DIEP is a myocutaneous flap first described in the early 1990s and popularized by Blondeel that ultimately replaced the pedicled and free transverse rectus abdominis muscle flap (TRAM) [10,11,12]. The DIEP gained dominance for abdominal-based reconstruction due to a lower donor site morbidity, reduced risk of hernia/bulge, bilateral capability, and cosmesis with the appearance of abdominoplasty. The DIEP is the gold standard today for fully autologous breast reconstruction, particularly useful in the setting of radiated fields. Additional more exotic options have arisen, including the superior gluteal artery perforator (SGAP) flap, profunda artery perforator flap (PAP), superficial inferior epigastric perforator (SIEP), transverse upper gracilis (TUG) flap, and the lumbar artery perforator (LAP) flap, although the DIEP is the most prolific option for total autologous reconstruction [13].

Endoscopic flap harvest techniques have been developed to reduce morbidity and incisional length but are considered very technically difficult, with limitations in instrument mobility and two-dimensional visualization, and have not gained much popularity as a result of technical challenges [14,15,16]. The robotic approach allows for a similar minimally invasive approach with greater dexterity and potential to overcome the laparoscopic technical limitations [17].

The National Aeronautics and Space Administration (NASA) and the United States military initially invested in robotic-assisted techniques in hopes of creating a “telepresence” surgery where the surgeon could be in a different place from the patient. However, due to technological and practical constraints, this effort was transferred to Intuitive Surgical for their da Vinci platform. Robotic-assisted surgery was first approved by the FDA in 2000 for the da Vinci Surgical System and has since revolutionized modern surgery [18]. General advantages of robotic surgery include tremor elimination, enhanced precision, improved ergonomic positioning, and 3D magnification. Robotic surgery has been found to reduce morbidity for patients, with decreased intraoperative blood loss, shorter hospital stays, reduced pain, and faster recovery [19,20,21]. The da Vinci robotic platform’s use has expanded from laparoscopic abdominal/pelvic-based surgeries to the chest, head/neck, and soft tissues [22]. Robotics in plastic/reconstructive surgery is a relatively new concept with a slower embrace due to large spatial tissue defects and the vast majority of open and non-cavitary procedures. The initial utilization of robotic assistance in breast reconstruction was for the dissection of the internal mammary vessels followed by the traditional free flap approach in 2006 [23]. This particular technique offered increased length for the anastomosis but did have a high hematoma complication risk. Factors precluding robotic use include dense abdominal adhesions requiring open surgery, unfavorable perforator anatomy, bleeding, or other intraoperative causes that obstruct camera view.

The robot was introduced to breast reconstruction in 2012 through a novel study that demonstrated improved outcomes, paving the way for further expansion of robotic techniques in the field [17]. A review of most contemporary literature reveals two promising techniques for robotic-assisted breast reconstruction: the latissimus dorsi (LD) flap and the deep inferior epigastric perforator (DIEP) flap [24,25]. Furthermore, mastectomy techniques have also been developed using the robot platform to perform minimally invasive quadrantectomy, partial mastectomy and nipple-sparing mastectomy [26]. Advances in the da Vinci Sp (single port) robotic platform that have allowed for advances culminating in true single-incision mastectomy and LD reconstruction [27]. Overall, minimally invasive techniques are appealing for lower donor site morbidity, lower hospital length of stay, and improved cosmesis at the expense of operative time and surgeon learning curve.

## 2. Materials and Methods

**Figure 1:**
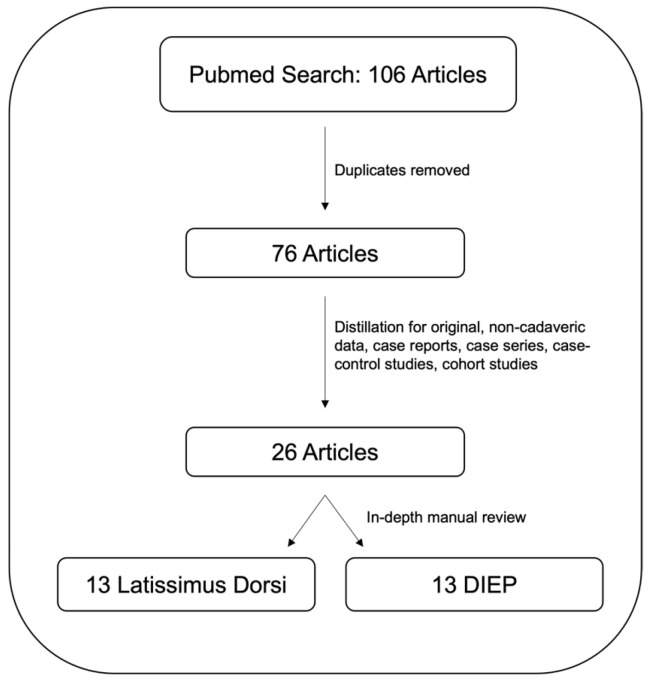
PubMed database search with the MESH term “Robotic breast reconstruction” combined with the Boolean operators AND “Flap” AND “Latissimus”, OR “DIEP” to refine the search, followed by application of inclusion criteria and manual review.

A literature review was conducted using a PubMed database search with the MESH terms “Robotic breast reconstruction” combined with the Boolean operators AND “Flap” AND “Latissimus”, OR “DIEP” to refine the search. The search was limited to studies published in English and published before November 2024. A total of 106 results were generated, which were then manually reviewed. Duplicates and non-contributory articles were removed with a remaining 73 articles. These were further manually reviewed for a total of 26 articles reporting operative data and summarized to provide a comprehensive overview of the current status, techniques, variations, and outcomes of robotic breast reconstruction (see Figure 1). Cadaveric studies were excluded. Of the studies fulfilling the inclusion criteria, there were case series, case reports, and retrospective case–control/cohort reviews. Internal review regarding ethics of the study was not necessary as all information was gathered from previously published and publicly available sources.

## 3. Results

### 3.1. Robotic-Assisted Latissimus Dorsi (LD) Flap Breast Reconstruction

The LD pedicled flap is a well-established option for breast reconstruction, particularly for patients not eligible for an abdominal-based autologous reconstruction [28]. LD flaps are often selected for patients with smaller body habitus and have an intact thoracodorsal blood supply, which may be injured during the index oncologic operation, particularly if there was an axillary lymph node dissection [29]. Traditionally, the open LD flap requires a long incision, 15–45 cm in length, where the LD is carefully dissected, paying special attention to the pedicle thoracodorsal artery and vein. Alternative endoscopic harvest techniques have been described with similar complication rates, improved incisional length, and post-operative pain, but they also face technical challenges with limited mobility and difficult dissection [14,15]. The pedicled latissimus flap is elevated and tunneled under the skin to the mastectomy site to provide implant coverage and inframammary fold support. Multiple variations of the robotic LD flap harvest exist with variations in port placement, insufflation, and combination with robotic nipple-sparing mastectomy. The major advantages of robotic LD harvest are that it allows for a smaller incision, improved cosmesis, and decreased hospital length of stay. Table 1 displays inclusion criteria sources with pertinent outcome data. 

The first robotic-assisted LD flaps were completed by Selber et al. in 2012 in a case series (n = 8) with promising results. The robotic harvest technique employed a short axillary incision for pedicle dissection and two to three additional ports with 10 mmHg insufflation for robotic instrumentation as opposed to a 15–45 cm incision in the traditional open technique. The robot was used to elevate the LD from the proximal port sites and release it from its distal origin and insertion sites with drain placement at the port sites. In the case series, robotic time decreased from 2 h 35 min to 1 h 5 min from the first to the last patient (average: 1 h 51 min). One reported complication of contralateral transient radial nerve palsy related to positioning. No donor site hematoma/seroma or skin complications were noted [30].

Clemens et al. published the next major robotic LD study in a 2014 retrospective cohort study based on Selber’s technique which evaluated 12 robotic LD flaps compared to 76 total open technique (TOT) flap harvests in patients undergoing treatment with immediate tissue expansion, similar levels of radiation, and timing for delayed reconstruction. Robotic flap harvest had a decreased complication rate, 16.7% robotic vs. 37.5% TOT (*p* = 0.31), including decreased seroma (10.9% versus 8.3%) and infection (14.1 versus 8.3%), delayed wound healing (7.8% versus 0), and capsular contracture (4.7% vs. 0), although not statistically significant, likely due to small sample size. Robotic LD had a longer operative time on average (1 h 32 min) compared to 58 min for the open technique. A decreased average hospital length of stay of 2.7 (2–3) was observed in the robotic LD group vs. 3.4 (3–6) in the open group. At one-year follow-up, the robotic cohort had similar surgical complication rates, including seroma, capsular contracture, and wound healing [31]. 

A trans-axillary gasless robotic LD harvest was described by Chung et al. in 2015. A long retractor and two additional port sites were used in twelve patients who underwent robotic LD flap reconstruction, three delayed breast reconstructions, four immediate reconstructions with nipple-sparing mastectomy, and five patients undergoing chest wall reconstruction for Poland syndrome. The average robotic time was 85.8 min; the average operative time was 400.4 min; the average docking time and robotic time decreased throughout the case series. This study also reported survey-based patient satisfaction scores averaging 9.2/10 for breast symmetry, 9.9/10 for scar appearance, and 9.6/10 for general aesthetic over a 15.7 month follow-up period [28].

The first case report for immediate robotic LD harvest following robotic quadrantectomy was performed in 2018 by Lai et al. for a patient with T3N1M0 breast cancer who underwent neoadjuvant chemotherapy followed by robotic left upper lateral quadrantectomy and robotic latissimus flap harvest through an axillary incision and two port incisions. The robotic time was 267 min but had decreased to 97 and 90 min during subsequent author operations. This case was complicated by donor site seroma that resolved with serial outpatient aspiration [32].

A series of 23 robotic-nipple sparing mastectomy procedures with robotic LD harvest were performed by Houvenaeghel et al. in 2019: 17 with LD flap alone and 6 with LD flap and implant. While this study focused primarily on robotic nipple-sparing mastectomy and differences in robotic mastectomy dissection technique, it did highlight a learning curve of 10–11 operations to establish a consistent learning curve for robotic nipple-sparing mastectomy and LD reconstruction. This was followed by a separate analysis of 80 robotic LD flaps with 71 immediate robotic LD flap reconstructions and 8 delayed reconstructions. This series was performed with a 4–7 cm axillary incision varying in habitus, gel port, and two separate robotic trocars at 7 mmHg insufflation. The median surgery time was 301 min, with a complication rate of 41%, with 29 patients developing donor site seroma and 1 patient who developed a donor site hematoma requiring re-operation. The average hospital stay was 4 days [33,34,35,36].

Houvenaeghel et al. published another study comparing open LD harvest and robotic LD harvest in skin-sparing mastectomy with immediate reconstruction (46 robot, 16 with implant; 59 open, 7 with implant). The open LD group had a higher rate of neoadjuvant chemotherapy (50.8% vs. 19.6%, *p* = 0.001) and previous radiation (66.1% vs. 30.5%, *p* < 0.0001). A higher rate of sentinel node biopsy was observed in the robotic group (53.5% vs. 22% for open, *p* = 0.016). The median anesthesia/surgical time was higher for the robotic group at 356.9/290.5 min compared to 327.5/259.7 min for the open group. Breast implants with LD were associated with longer operative times and a slightly longer hospital stay. There was a similar complication rate but a difference in the severity of complications upon univariate analysis, with higher severity in the robotic cohort requiring re-operation (two vs. one for open) [37].

Moon et al. published a 2020 case series of 21 robotic LD flaps performed for Poland syndrome. This was the first case series dedicated to Poland syndrome reconstruction (5–6 cm axillary incision with three robotic ports and an axillary retractor, lateral decubitus positioning, average hospital length of stay: 7 days; low complication rate of 19% with 4x seromas and 1 axillary wound complication). Fifteen patients required re-operation (seven breast augmentations, eight lipofilling procedures, four contralateral breast surgeries [38]).

Wincour et al. published a 2020 retrospective cohort study in which 52 LD flaps were evaluated (25 robotic, 27 open). Longer operative times were observed with robotic technique, averaging 388 (245–519) vs. 311 (171–488) for open. Similar complication rates were reported, although there was a notably higher rate of seroma (19% vs. 0% open). A shorter hospital length of stay was observed in the robotic group, with an average of 2 vs. 3 days. The authors also found a lower but not statistically significant opioid use in the robotic group [39].

Joo et al. published a 2021 case report using single-incision robotic NSM and robotic LD using the da Vinci SP system, involving a 4.5 cm axillary incision through which both procedures were performed. Comparable time to other robotic LD flaps was observed and without additional ports (100 min console time, 15 min docking time, no complications, and above-average breast-Q patient satisfaction score (67 vs. 55/100 author average) [27]).

Cheon et al. published a 2022 retrospective case series from S. Korea in an overview of 10 years of robotic LD flap harvest and evolution from DaVinci Si 2012–2014 to Xi 2014–2018 to SP 2018–2021. The technique had changed drastically, from the Si and Xi multiport platforms to the single-port system with a retractor and three additional port placements to a single port with insufflation. Si involved one infection (7.7%), five seroma (38.5%), two cases of donor site morbidity (15.4%), and eight wound problems (61.5%). Xi involved two infections (11.1%), six seroma (33.3%), one flap loss (5.6%), four donor site morbidities (22.2%), and ten wound-related problems (55.6%). Sp involved one hematoma (10%), two seroma (20%), two donor site morbidities (20%), and two wound-related problems (20%). There was no significant difference in complication rate between groups, although it is notable that the SP group had a lower wound complication rate and seroma rate. The study applied aesthetic scores from pre- and post-operative photographs from four plastic surgeons, with no significant difference between the groups [40]. 

Hwang et al. published a 2022 case series of three patients with Poland syndrome who underwent chest wall reconstruction with the da Vinci SP system. Two males and one female had an implant and contralateral robotic augmentation (no reported complications, 5 cm axillary incision, greatly reduced robotic docking time of 8.67 min (4–14) compared to previously reported average of 23–55 min [41]). 

Eo et al. published a 2024 Korean single-institution first-of-its-kind study comparing robotic, endoscopic and open LD reconstruction following partial mastectomy. In total, 17 endoscopic LD, 20 robotic, and 20 open cases were performed. The robotic technique used an axillary incision and a single inferior port using the da Vinci Si system. There was a significantly longer average operative time for robotic (394.4) compared to endoscopic (316.6) and open (279.8) techniques, with a significantly higher robotic time than endoscopic operative time (robot: 75.7 ± 30.7; endoscopic: 34.5 ± 12.9, *p* < 0.001). There was no significant difference in opioid use among techniques, no significant difference in hospital length of stay, and no significant difference in complication rates. There was a significant difference in patient satisfaction using patient survey modified BREAST-Q scores, with higher rates of patient satisfaction for robotic/endoscopic LD compared to open for posterior scars and overall satisfaction, but no difference between robotic and endoscopic techniques [16].

### 3.2. Robotic-Assisted Deep Inferior Epigastric Perforator (DIEP) Flap Breast Reconstruction

DIEP flap breast reconstruction has emerged as the gold standard for autologous flap-based reconstruction. The DIEP flap is based on perforating vessels from the deep inferior epigastric artery which provides blood supply extending from the external iliac artery/vein to the abdominal wall musculature, subcutaneous adipose, and skin. The DIEP is anastomosed to the internal mammary artery/vein in the chest or to the thoracodorsal vessels laterally to achieve breast reconstruction. Robotic assistance has been applied in dissecting the recipient’s internal mammary vessels as well as the establishment of the robotic DIEP harvest in dissecting the vascular pedicle [23]. One of the major shortcomings in traditional DIEP harvest is abdominal rectus muscle and anterior fascia damage to isolate the vascular pedicle leading to increased rates of hernia and abdominal bulging despite fastidious primary closure. Below the arcuate line (~1/3 distance from umbilicus to pubis), there is no posterior rectus fascia, and the anterior fascia closure is the only abdominal strength layer; 71% of robotic DIEP patients had an anterior fascial incision contained above the arcuate line reported by Kurlander et al. [42]. There is a limited patient population that qualifies for robotic DIEP requiring a short intramuscular perforator course, ideally above the arcuate line, and two maximum perforators. Rates of hernia/abdominal bulge have been reported to be between 0.18% and 1.26% with associations with prior abdominal surgery, pregnancy history, smoking and age [43]. The robotic DIEP harvest sets out to improve this complication rate. Two methods of minimally invasive DIEP harvest have been described, a trans-abdominal preperitoneal (TAP) approach by Gundlapalli [24] and Selber [44] and the total extraperitoneal (TEP) approach first described laparoscopically, the difference being, for the TAP approach, that the peritoneal cavity is entered and the vessels are dissected with a peritoneal flap, whereas the TEP approach uses insufflation between the peritoneum and abdominal wall to isolate and transect the vessels without entering the peritoneal cavity. Inclusion criteria for robotic DIEP flap sources listed in Table 2 and Table 3 with pertinent outcome data.

The first robotic DIEP harvest was described by Gundlapalli et al. in a 2018 case report. A robotic DIEP flap was performed in a patient with previous neoadjuvant chemotherapy, modified radical right mastectomy, and adjuvant radiation. The left hemiabdomen DIEP flap was elevated with a 1.5 cm fascial incision and 10 cm pedicle length with 20 min robotic docking time and 40 min of console time. The authors claim 40 min is comparable with open pedicle dissection. The pedicle was dissected using a TAP technique with robotic running suture closure of the peritoneum. Four robotic ports were placed on the contralateral anterior axillary line. No immediate complication occurred at either the flap or donor site or at 9-month follow-up. Cost analysis was USD 16,300 patient charge for robotic procedure and USD 14,800 for typical open procedure [24].

Selber published a technique article outlining trans-abdominal pre-peritoneal DIEP harvest (TAP). The described technique uses three ports on the contralateral side identical to robotic rectus muscle flap port placement and a reported 1.5–3.0 cm fascial incision and 10–15 cm of pedicle length. Large emphasis was placed on pre-operative planning with CTA and favorable perforator anatomy with short intramuscular course and 1–2 closely grouped perforators. The authors reported a subjective decrease in pain and hospital stay [44].

Choi et al. described the first extraperitoneal (TEP) DIEP harvest in 2021 utilizing the da Vinci SP system and placing the single robot port through the neo-umbilicus. This method conserves the posterior rectus sheath and does not enter the peritoneal cavity, reducing the risks associated with abdominal surgery (bowel injury, adhesions, etc.); 17 of 21 DIEP patients were treated with this method from 9/2019 to 8/2020, with inclusion criteria being an intramuscular vascular segment <5 cm. The described technique involved a 1.5 cm incision on the ipsilateral linea semilunaris for blunt finger dissection in the extraperitoneal space followed by 2 × 2 cm cross incision at the new umbilicus for the robot port. Muscular pedicle dissection was performed open, followed by robotic dissection to the origin at the external iliac. The average robotic time was 65 ± 33 min. The average surgery time was 487 ± 93 min. Metrics on complication rate, hospital length of stay, comparison to open technique, or follow-up were not published [45].

Kurlander et al. (including Selber) published a retrospective study analyzing pre-operative CT angiogram for robotic DIEP eligibility from 2017 to 2021. CT angiograms were reviewed for 49 patients (98 hemiabdomens). Inadequate or non-perforators were identified on CTA in 18% of hemiabdomens. Mean predicted robotic and open DIEP fascial incisions were 3.1 cm and 12.2 cm, respectively, giving the robotic approach to fascial incision a benefit of 9.1 cm (*p* < 0.001). The predicted robotic incision avoided crossing the arcuate line in 71% of hemiabdomens. Thirteen patients (28%) underwent robotic DIEP harvest. The actual robotic fascial incision length averaged 3.5 cm, which was not significantly different from the mean predicted fascial incision length (*p* = 0.374). Robotic DIEP flaps had fewer perforators (1.8 versus 2.6, *p* = 0.058); 27% of patients analyzed underwent robotic DIEP, and the authors proposed that more patients were eligible for the procedure than those receiving it. A large emphasis was also placed on having an anterior fascial incision above the arcuate line to minimize the risk of hernia with an intact posterior rectus sheath [42].

Piper et al. published a 2021 four-patient case series from UCSF for TAP robotic DIEPs. Notably, the included patients also underwent another robotic intra-abdominal procedure at the same time as bilateral DIEP harvest and breast reconstruction, minimizing the need for a second operation. The first patient also underwent gastric resection for a benign mass. The pedicle dissection time was 38 min and the hospital stay was 10 days. The second patient was BRCA2-positive and underwent prophylactic oophorectomy during the same operation. The DIEP pedicle dissection time was 42 min; she was discharged on hospital day 2. The third patient was BRCA2-positive and underwent robotic hysterectomy and bilateral salpingo-oophorectomy. The pedicle dissection time was 52 min and hospital stay was 2 days. The fourth patient BRCA2-positive and underwent concurrent hysterectomy and bilateral salpingo-oophorectomy. The pedicle dissection time was 48 min and she was discharged after 2 days of hospitalization. Notably, this was the first time a bilateral DIEP flap harvest was performed with another intra-abdominal procedure [46].

Shakir et al. performed a 2021 retrospective cohort analysis of endoscopic DIEP compared to laparoscopic TEP and robotic TAP; 94 patients underwent endoscopic DIEP harvest, 38 underwent TEP LAP harvest, and 3 patients underwent robotic bilateral reconstruction. All three patients subject to the robotic technique underwent synchronous additional intra-abdominal procedures at the time of operation; two abdominal hysterectomies and bilateral salpingo-oophorectomies and one partial gastrectomy. Average operative times varied at 249 min for a unilateral endoscopic DIEP, 535 min for bilateral endoscopic harvest, 335 min for unilateral TEP laparoscopic harvest, 453 for bilateral TEP laparoscopic harvest, and 535 for bilateral TAP robotic harvest; 5/142 endoscopic and 2/67 TEP LAP patients had perforator injuries during dissection, with a rate of 0/3 in the robotic cohort. The endoscopic cohort had one arterial thrombus and three cases of venous congestion and the TEP laparoscopic cohort had one case of venous congestion. No robotic complications were reported. Cost analysis was performed with an additional increase of approximately ∼USD 234 per case of disposable cost endoscopically, ∼USD 495 for TEP laparoscopic harvest, and ∼USD 1487 for TAP robotic harvest. The average length of stay significantly differed, as subjects in the TAP robotic cohort remained in the hospital for an average of 4.7 days versus 2.8 and 2.5 days in the endoscopic and TEP laparoscopic cohorts. This increased length of stay in the robotic cohort is skewed by additional procedures performed for robotic-cohort patients. Overall, the authors prefer the laparoscopic TEP technique, and the study has confounding variables as the all patients in the robotic DIEP cohort also underwent concurrent intra-abdominal procedures [47].

Wittessaele et al. published a 2021 case series of 10 TAP robotic DIEP flaps. All flaps were unilateral with ipsilateral docking of the robot and three ports. The average docking time was 27.5 min (16–40), the average fascial incision was 3 cm, the average console time was 86 min (52–162), and the surgery duration was 479 (409–552). Patients were followed up at 2 and 6 weeks. No intra-abdominal complications or flap loss was observed. One hematoma occurred at the recipient site requiring evacuation. The authors are supportive of robotic surgery, had no prior robotic experience, and are confident that with additional practice, the operative time will be comparable to open surgery with considerably smaller fascial defects [48].

Lee et al. published a 2022 retrospective cohort study comparing 186 open unilateral DEIP flaps to 21 unilateral TEP single-port robotic flaps. The technique was similar to that previously described with a single robotic port at the neo-umbilicus. There was found to be a longer operative time in the robotic cohort (509 ± 71 vs. 438 ± 83) but significantly decreased length of stay (7.92 ± 1.2 vs. 8.77 ± 1.74), improved pain scores, less narcotic use and significantly higher scores for post-operative psychosocial well-being (*p* = 0.007), physical well-being of the chest (*p* = 0.028), and physical well-being of the abdomen (*p* = 0.02). Complications were reported: 5.3% (1) flap loss and 5.3% (1) fat necrosis in the robotic cohort, and 2.2% flap loss (4), 1.1% (2) fat necrosis, 1.1% (2) seroma, and 6.5% (12) donor site wound problems in the open cohort [49].

Jung et al. published a 2022 case report of a patient that underwent robotic nipple-sparing mastectomy with immediate single-port TEP robotic DIEP. No complications, 7-day hospital stay and 7-month follow-up were reported. This was the first report of a fully robotic resection and immediate DIEP reconstruction using the da Vinci SP system [50].

Bishop et al. published a 2022, 21-patient case series on multiport TAP robotic DIEP reconstruction; 10% of patients had a prior intra-abdominal surgery. The average fascial incision was 3.6 ± 1.6 with an average pedicle length of 13.3 ± 1, console time of 45 ± 9 min, and total surgery duration of 425 ± 70 (unilateral) and 511 ± 67 (bilateral). The average hospital stay was 3.8 days ± 0.9. The average follow-up was 5 months, with 31.3% (five) surgical site occurrence, one wound healing complication, and no flap loss or hernia/bulging. Post-operative pain analysis was performed in which five patients had bilateral flaps, one harvested robotically and the other open, and were blinded to which side was harvested robotically. Four in five patients reported less pain on the robotic side with bilateral TAP blocks. Analysis of CTA’s positive predictability of fascial incision was 86% accurate within 2 cm/75% standard difference in predicted fascial incision from CT scan, validating CTA as a reliable pre-operative planning modality for robotic DIEP candidacy [51].

Tsai et al. published a 2023 retrospective cohort study comparing 13 (11 unilateral, 2 bilateral) robotic DIEP flaps to 86 (62 unilateral, 24 bilateral) open DIEP flaps. Robotic flaps were harvested in multiport TAP fashion with novel port placement described with a supra-umbilical camera port and bilateral instrument ports 10 cm from the midline at the corners of the skin incision near the linea semilunaris. This port placement does not require re-docking for contralateral pedicle dissection for bilateral DIEP flaps. Pre-operative imaging with CTA was used and the criterion for robotic candidacy was <2.5 cm intramuscular pedicle course. Significantly shorter fascial incision was reported at 2.7 ± 1.1 cm for robotic vs. 8.1 ± 1.7cm for open (*p* < 0.0001). Robotic time was reported to be 53 ± 13 cm for pedicle dissection and 22 ± 3.5 cm for peritoneal closure. Approximately 100 additional minutes of operating time were required for robotic DIEP with an approximate cost increase of USD 3500 in robotic instruments and disposables. One minor wound healing complication was reported in robotic cohort and two minor wound healing complications were reported in the open cohort. Both cohorts had a 3-day ICU admission post-operatively for flap monitoring, before mobilization on hospital day 4. Follow-up was carried out at 2, 4, and 12 weeks. No statistical difference in pain scores between robotic and open cohorts on hospital days 1–3 was reported [52].

Murariu et al. published a 2024 retrospective cohort study consisting of 46 bilateral robotic DIEP flaps (23 patients) outlining a TAP multiport robotic flap harvest technique similar to that described by Tsai. Three ports (supraumbilical camera and bilateral instrument ports) were used in conjunction with the first reported use of ICG dye in robotic DIEP for pedicle identification and dissection, with an average fascial incision of 4.1 cm, average pedicle length of 12.8, average console time of 139 min, average OR time of 739 min, and average hospital stay of 3.9 days with 90-day follow-up. No flap harvest/pedicle injuries occurred; there was one patient that had partial flap necrosis requiring a revision surgery and one patient with an abdominal wound complication. No cases of post-operative hernia or bulging occurred. There were two instances where the case was converted to traditional technique; one was due to dense intraperitoneal adhesions preventing safe identification and dissection robotically, and one had a prior hematoma following C-section obscuring the anatomy and requiring traditional dissection on one side. Overall, this study establishes multiport TAP robotic DIEP as a valid technique to minimize fascial incisional length. The authors recommend assistance with a general surgeon for the robotic portion until being comfortable with robotic pedicle dissection and the novel use of indocyanine green dye (ICG) fluorescence to assist in the identification of pedicle vessels [53,54].

Moreira et al. published a 2024 retrospective cohort study evaluating the learning curve for a plastic surgeon compared to a robotics-certified general surgeon in TAP multiport robotic DIEP pedicle dissection. Using the cumulative sum method, there were 44 flap dissections performed, 27 by the plastic surgeon and 17 by the general surgeon. There was no significant difference in dissection time between the GS (34.8 min) and PS (44.6 min) (*p* = 0.366). Both surgeons saw a decrease in dissection time with the increasing number of cases. The cumulative sum peaked at patient 9 for the PS and patient 5 for the GS, after which dissection time consistently decreased to significantly faster dissection times at the end of the study period. Seven patients had a bilateral procedure where one surgeon performed the dissection on each side, but the sample size was too small to be amenable for meaningful analysis. There were no intra-abdominal injuries, pedicle injuries, conversions to open surgery, flap losses or long-term complications of hernia/bulge after 1 year of follow-up. After 10 flap harvests, comparable operative times between plastic and general surgeons were achieved. Overall, this study shows the feasibility of robotics in plastic surgery with a short learning curve for robotic training, high level of safety and improved donor site morbidity after approximately 10 cases [55].

Moreira et al. published a 2024 follow-up retrospective cohort study to match Murariu’s study comparing multiport TAPP robotic DIEP to open technique. In total, 47 patients were included (48 standard DIEP flaps, 46 robotic DIEP flaps) with similar patient characteristics and prior abdominal surgical histories. Fascial incision length in the robotic DIEP group was shorter (4.1 vs. 11.7 cm, *p* < 0.001) with no significant difference in pedicle length (*p* = 0.238). Mesh reinforcement of the abdominal wall was used in 13/24 standard DIEPs, with none in robotic DIEP patients (*p* < 0.001). Operative time was longer in the robotic DIEP cohort (739 vs. 630 min, *p* = 0.013), although sub-analysis showed no significant difference in the operative times of the second half of the robotic cohort, attributable to robotic experience and learning curve. The average robotic dissection time was 135 min, which decreased significantly with the surgeon’s experience. There were no intraoperative complications in the robotic cohort. Hospital length of stay was shorter with robotic DIEP, but not statistically significant (3.9 vs. 4.3 days, *p* = 0.157). Overall, the study highlights the viability of robotic DIEP harvest with decreased fascial incision, similar immediate complication rates, decreased need for anterior rectus mesh use, and decreased robotic time with practice [56].

## 4. Discussion

The latissimus dorsi and DIEP are two flaps currently being harvested for breast reconstruction with robotic technology. Various techniques have been described using multiport vs. single-port systems, variance of port placement, retraction vs. insufflation, and the TAP vs. TEP approach. Overall, the majority of research has been published from single institutions and has found improved cosmesis/patient satisfaction for robotic latissimus harvest with considerably smaller skin incision while retaining similar flap efficacy and complication rates. Robotic technology allows for the possibility of a single axillary incision for robotic nipple-sparing mastectomy and reconstruction with ipsilateral LD. For DIEP flaps, robotic harvest has shown consistently smaller fascial incisions during pedicle dissection with similar complication rates to the open technique. There is a limited patient population that qualifies for robotic DIEP, requiring a short intramuscular perforator course below the arcuate line, sparing the posterior abdominal fascia, and two maximum perforators. Long-term data for reduction abdominal bulge and hernia are yet to be seen, but preliminary data are promising. Robotic TAP DIEP harvest can also be combined with other robotic intra-abdominal procedures at the same time if needed. The da Vinci SP (single port) system has shown great promise in TEP DIEP harvest, sparing intra-abdominal complications with small fascial incisions, although it has limited approval for use in the US. Overall, robotic flap harvest has been shown to have similar efficacy to traditional techniques, similar immediate complications, improved cosmesis, slightly higher cost, and increased operating time that decreases with surgeon experience/learning curve, with long-term complications yet to be seen.

### Limitations

There are no standardized training programs for robotic plastic surgery, and robotic training is currently not part of the plastic surgery residency curriculum. Robotic flap harvest is not routinely performed on a large scale and has a wide variance in techniques, leading to the slow rate of adoption and lack of large-scale outcome data. Most available studies are retrospective without any blinded large-scale multi-center randomized control trials. Potential bias is introduced based on recall of data and variance in technique between surgeon, institution, and robotic platform. There is lack of standardization in what constitutes a complication, minor vs. major, how they are reported, and varying follow-up. Further possible bias is introduced with a large initial surgeon investment to perform robotic flap surgery, and those performing/reporting outcomes are likely to favor robotics. Barriers to starting robotic surgery include expensive starting costs with equipment and the need for robotic training and capable facilities. Limitations of our study are that conclusions can be only made from existing research, and a higher level of evidence is required for optimal evaluation of robotic breast reconstructive techniques. This review does not incorporate ongoing research, only published publicly available works. The author’s institution performs robotic flap surgery for pelvic reconstruction, but not for breast reconstruction, and is unbiased in this subset of the field.

With the benefits of minimally invasive mastectomy and breast reconstruction, the ability to perform robotic mastectomy and reconstruction is becoming more feasible with the great appeal of single-lateral-incision nipple-sparing mastectomy and LD reconstruction. Robotic DIEP harvest theoretically has a lower risk of abdominal hernia/bulge complications, but long-term data are not yet available. One of the major future directions for robotics in plastic reconstructive surgery is robotic assistance in microsurgical and supermicrosurgical anastomoses with the ability to scale down movement and filter out physiologic human tremors. Future advances in robotic reconstructive surgery are likely going to be guided by innovative systems, such as the da Vinci SP (single port) system, Microsure MUSA, the MMI Symani microsurgery robotic system, and the BHS Roboticscope digital microscope. As robotics becomes more pervasive, utility in plastic surgery will become more common and an increased level of research is to be expected, with larger studies and long-term outcome data focused on incisional and technical complications/benefits. Robotic systems have been used for head and neck reconstruction anastomoses with limited space, peripheral nerve/brachial plexus repair, and lymphaticovenous bypass procedures for lymphedema treatment [57]. The next forefront in robotic microsurgery/reconstruction is in its infancy with the use of novel systems to perform microsurgery and supermicrosurgery <1 mm on blood vessels, lymphatics, and nerves. The Symani system is starting to be used for these applications with movement scaling 7–20 fold and tremor elimination; robotic technology is advancing to a point where it will outperform even the most skilled surgeons based on physiologic limitations [58,59,60]. The Symani system has started to be used in Europe over the last few years and recently gained FDA approval in the US in April 2024. It has been shown to have similar efficacy to hand-sewn anastomoses in emergent hand reconstruction with promising results after a learning curve of 10 cases with a 30% increase in speed [61]. The MUSA system has been used for supermicrosurgical lymphaticovenous bypass to treat breast cancer-associated lymphedema and shown to be non-inferior to manual bypass at one-year follow-up [62]. There is great potential for the use of new robotic systems, taking robotic reconstructive surgery to new heights.

Future directions include an increased level of evidence in investigating robotic breast reconstruction with blinded, prospective, multi-center randomized controlled trials; long-term follow-up for outcomes of robotic techniques, particularly with regard to robotic DIEP flap abdominal hernia/bulge rates; and an increased prevalence of the use of existing robotic platforms such as da Vinci, da Vinci SP, and novel microsurgical/supermicrosurgical robotic technology with platforms like Symani and Roboticscope.

## 5. Conclusions

Robotic plastic surgery and flap harvesting for breast reconstruction is still in its early stages. There is great promise shown in reducing donor site morbidity, complications, and incision length while retaining a similar or improved degree of cosmesis, lower complication rate, and quality in the result at the expense of cost and operative time. The two flaps being used for breast reconstruction robotically are the latissimus dorsi and the DIEP flap. The major advantage to the LD is a smaller incision leading to a lower wound complication rate and improved cosmesis. The major advantage of a robotic DIEP is a more precise and less traumatic harvest of the inferior epigastric perforator vessels, minimizing the anterior abdominal fascial defect, potentially leading to a reduction in hernia and abdominal bulging post-operatively due to preservation of the fascia and anterior rectus sheath.

## Figures and Tables

**Table 1:** Robotic latissimus dorsi source flap volume, incision, console/OR time, hospital stay, complication rate and patient satisfaction.

Source	Number of Robotic Flaps	Number of Open Flaps	Incisional Length (Robotic) (Avg)	Console Time (Avg)	Surgery Duration (Robotic) (Avg)	Surgery Duration (Open) (Avg)	Hospital Stay (Robot) (Avg Days)	Hospital Stay (Open) (Avg Days)	Complication Rate (Robot)	Complication Rate (Open)	Patient Satisfaction Analysis
Selber 2012 Case series [30]	8	0	5 cm	111 min	NR	NR	NR	NR	1: Transient peripheral neuropathy	NR	NR
Clemens 2014 Retrospective cohort study [31]	12	64	5 cm	NR	NR	NR	2.7 Days	3.4 Days	16.7% Total (*p* = 0.31), Seroma 8.3%, Infection 14.1%, Re-operation 8.3%	Seroma 8.9%, Delayed healing 7.8%, Infection 8.3%, re-operation 12.5%, Capsular contracture 4.7%	NR
Chung 2015 Retrospective case series [28]	12	0	5.5 cm	85.8 min	NR	NR	NR	NR	One patient dissatisfied with upper inner quadrant appearance, resolved with 3x outpatient fat grafting	NR	9.2 for breast symmetry, 9.9 for scar and 9.6 for general aesthetic result out of 10
Lai 2018 Case report [32]	1	0	4 cm	97 min	NR	NR	NR	NR	1× seroma; 100%	NR	NR
Houvenaeghel 2019 Prospective [33,34,35,36]	80	0	5.5 cm	NR	310 min	NR	4 Days	NR	41.10%. Grade 1: 29 seromas. Grade 3: 1 = bleeding requiring re-operation	NR	NR
Houvenaeghel 2020 Prospective case–control [37]	46	59	5. 5 cm	NR	290.5 min	259.7 min open *p* = 0.016	4.3 Days	3.8 Days *p* = 0.089	45.70% Total. 12 Seroma, 1 bleeding, 2 dorsal infection, 2 re-operation	62.70% Total. 23 seroma, 2 bleeding, 1 re-operation	NR
Moon 2020 Prospective case series [38]	21	0	5.5 cm	58 min	295 min	NR	7 Days	NR	4x Seromas all resolved w/aspiration, 1× axillary wound complication	NR	improvement in chest deformity, satisfaction with overall outcomes, chest symmetry, and scars were 4.80, 4.72, 4.18, and 4.87 out of 5
Wincour 2020 Retrospective cohort study [39]	25	27	5.5 cm	NR	388 min	311 min	2 Days	3 Days	16% Seroma	3% Hematoma	NR
Joo 2021 Case report SP [27]	1	0	4.5 cm	100 min	328 min	NR	6 Days	NR	0%	NR	Breast-Q 67/100. Avg test score is 55
Cheon 2022 Retrospective cohort series SP [40]	41 Total: 13S, 18Xi 10Sp	0	4.5 cm	Si 85 min, Xi 64 min, SP 49 min, Total 70 min	Si 399 min, Xi 451 min, SP 496 min, total 439 min	NR	Si 12.6, Xi 9.7, Sp 7, Total 9.0	NR	Si: 1 infection (7.7%), 5 seroma (38.5%), 2 donor site morbidity (15.4%), 8 wound problems (61.5%). Xi: 2 infections (11.1%), 6 seroma (33.3%), 1 flap loss (5.6%), 4 donor site morbidities (22.2%), 10 wound-related problems (55.6%). Sp: 1 hematoma (10%), 2 seroma (20%), 2 donor site morbidities (20%), 2 wound-related problems (20%)	NR	Harris breast scale, no significant difference among groups but Sp patients did have higher scores
Hwang 2022 Retrospective case series SP [41]	3	0	5 cm	NR	449 min	NR	NR	NR	0%	NR	NR
Eo 2023 Prospective Cohort study (Also included 17 endoscopically harvested LD) [16]	20	20	4 cm	75.7 min, Endoscopic 34.5 min *p* < 0.001	394.4 min, 316.6 min endoscopic	279 min	10.2, 10.8 endoscopic	9.75	15% robotic, 11.8% endoscopic; 3 seroma (robotic), 2 seroma (endoscopic), 1 wound dehiscence each	20% Total; 3 Seroma (15%), 1 wound dehischence (5%).	Modified BREAST-Q: Significantly lower satisfaction with scar on the back and overall between open vs. robotic/endoscopic. No significant difference between endoscopic and robotic.

NR stands for not reported. Evolution of robotic platform over time. SP stands for single port. Eo in 2023 also compared endoscopic harvest to robotic and open. Patient satisfaction metrics included when available.

**Table 2:** DIEP flap numbers, fascia incisional length, surgery duration, and follow-up interval.

Source	Number of Robotic Flaps	Number of Open Flaps	Fascial Incisional Length (Avg)	Surgery Duration (Avg)	Follow-Up Interval
Gundlapalli 2018 Case report TAP [24]	1	NR	1.5 cm	531 min	9 Mos
Selber 2021 Technique TAP [44]	NR	NR	1.5–3.0 cm	NR	NR
Choi 2021 Retrospective case series TEP [45]	17	4	1.5 cm	487 min	NR
Kurlander 2021 Retrospective case review TAP [42]	13	49	3.5 cm	NR	NR
Piper 2021 Case series TAP [46]	8	NR	NR	NR	NR
Shakir 2021 Retrospective cohort study TAP/TEP [47]	3	94 endoscopic, 38 TEP laparoscopic	2.0 TEPLAP, 4.5 endoscopic, NR robotic	249 min unilateral endoscopic, 535 min b/l endo, 335 min unilateral TEPLAP, 453 min b/l TEPLAP, 535 min TAPRAP	8 Mos
Wittesaele 2021 Case series TAP [48]	10	NR	3 cm	479 min	2 weeks and 6 weeks
Lee 2022 Retrospective cohort study TEP SP [49]	21	186	4.3	509 min robotic, 438 open	NR
Jung 2022 Case report TEP SP [50]	1	0	5 cm	NR	7 Mos
Bishop 2022 Case series TAP [51]	21	0	3.6 cm	425 min unilateral, 511 bilateral min	5 Mos
Tsai 2023 Retrospective cohort study TAP [52]	13 (11 unilateral 2 bilateral)	86 (62 unilateral 24 bilateral)	2.7 cm vs. 8.1 open (*p* < 0.0001)	~100 additional minutes robotic	3 days ICU, 2, 4, 12 weeks
Murariu 2024 Retrospective cohort study TAP [53,54]	46 (All bilateral robot)	NR	4.1 cm	739 min	90-day follow-up
Moreira 2024 Retrospective case series TAP [55,56]	46	48 (total flaps including bilateral)	4.1 cm robot vs. 11.7 cm open (*p* < 0.001)	739 min robotic, 683 min open	90 days, 284.6 days robot, 357.6 days open

NR stands for not reported. TEPLAP for Shakir is total extra-peritoneal laparoscopic technique.

**Table 3:** Robotic DIEP flap sources’ hospital stay, complications rates, patient satisfaction, and cost analysis.

Source	Hospital Stay (Robot) (Avg)	Hospital Stay (Open) (Avg)	Complication Rate (Robot)	Complication Rate (Open)	Patient Satisfaction	Cost Analysis
Gundlapalli 2018 Case report TAP [24]	NR	NR	0%	NR	NR	USD 16,300 robotic, USD 14,800 open
Selber 2021 Technique TAP [44]	NR	NR	NR	NR	NR	NR
Choi 2021 Retrospective case series TEP [45]	NR	NR	NR	NR	NR	NR
Piper 2021 Case series TAP [46]	4 Days	NR	NR	NR	NR	NR
Shakir 2021 Retrospective cohort study TAP/TEP [47]	4.7 Days	2.8 endoscopic 2.5 TEPLAP	NR	Pedicle injury flap loss ×1, 1× abdominal bulge endoscopic	NR	∼USD 234 per case of disposable cost, ∼USD 495 for TEP-LAP harvest, and ∼USD 1487 for RAP harvest.
Wittesaele 2021 Case series TAP [48]	NR	NR	1× Chest hematoma requiring drainage	NR	NR	NR
Lee 2022 Retrospective cohort study TEP SP [49]	7.92 Days	8.77	5.3% (1) Flap loss, 5.3% (1) Fat necrosis	2.2% Flap loss (4), 1.1% (2) fat necrosis, 1.1% (2) Seroma, 6.5% (12) Donor site wound problem	Breast-Q: 16 Robot, 59 Open. Robotic group had significantly higher scores for post-operative psychosocial well-being (*p* = 0.007), physical well-being of the chest (*p* = 0.028), and physical well-being of the abdomen (*p* = 0.02)	NR
Jung 2022 Case report TEP SP [50]	7 Days	NR	0%	NR	NR	NR
Bishop 2022 Case series TAP [51]	3.8 Days	NR	31.3% (5) Surgical site occurrence, 1× wound healing complication	NR	Five patients had bilateral flaps, one harvested robotically and the other open and patient was blinded. Four in five patients reported less pain on the robotic side.	NR
Tsai 2023 Retrospective cohort study TAP [52]	3 Days ICU	3 Days ICU	1/13 Wound healing minor complication	2/86: Minor wound complications	No significant difference in post-operative pain	~USD 3500 increase in robot instruments and disposables
Murariu 2024 Retrospective cohort study TAP [53,54]	3.9	NR	1× Partial flap necrosis, 1× abdominal wound complication	NR	NR	NR
Moreira 2024 Retrospective case series TAP [55,56]	3.9	4.3	1 Partial flap loss, 1 return to OR, 7 wound-related issues, 2 readmission for any reason	2 Abdominal bulges requiring intervention at revision surgery. 2 partial flap loss/return to OR, 3 readmissions any reason, 1 readmission for flap, 8 wound healing issues	NR	NR

NR stands for not reported. TEPLAP for Shakir is total extra-peritoneal laparoscopic technique and RAP is robotic abdominal pre-peritoneal harvest.

## Data Availability

Data available in a publicly accessible repository; the original data presented in the study are openly available within the PubMed database at [pubmed.ncbi.nlm.nih.gov] accessed on 24 November 2024. Individual source reference information is located within the citations of the reference section.

## Abbreviations

The following abbreviations are used in this manuscript:

LD	latissimus dorsi
DIEP	deep inferior epigastric perforator
TAP/TAPP	trans-abdominal pre-peritoneal
TEP	total extra-peritoneal
TRAM	transverse rectus abdominal muscle
UNLV	University of Nevada Las Vegas
CT	computed tomography
SP	single port
